# Efficacy of Fractionated Erbium Laser in Treating Radiation-Induced Dermal Atrophy: A Case Report

**DOI:** 10.7759/cureus.47339

**Published:** 2023-10-19

**Authors:** Ameije Ismaili, Jon Ward

**Affiliations:** 1 College of Osteopathic Medicine, Lake Erie College of Osteopathic Medicine, Bradenton, USA; 2 Dermatology and Mohs Surgery, Alabama College of Osteopathic Medicine, Panama City, USA

**Keywords:** radiation dermatitis, erbium:yag laser, squamous cell carcinoma in situ, superficial radiation therapy, dermal atrophy

## Abstract

Radiation dermatitis is a common side effect of radiotherapy in patients with acute and chronic changes affecting the skin. While acute changes occur within 90 days of radiation exposure, chronic changes manifest thereafter. This paper presents a case of a 70-year-old male with squamous cell carcinoma in situ (SCCIS) on the right zygoma who was treated with superficial radiation therapy (SRT), which resulted in a hypo-pigmented atrophic scar. The scar was successfully treated with a single session of Erbium:YAG laser therapy. The findings highlight the need for improved treatment options for radiation-induced skin changes and demonstrate the efficacy of fractionated laser therapy in addressing SRT-induced dermal atrophy.

## Introduction

Radiation dermatitis is a frequent adverse effect of radiotherapy observed in approximately 95% of patients undergoing treatment [1]. The severity of dermatologic effects varies based on clinical settings, with superficially targeted radiation, such as in skin cancer cases, resulting in more pronounced manifestations. Radiation-induced skin changes can be categorized into acute and chronic changes, with acute changes occurring within 90 days of radiation exposure and chronic changes emerging thereafter [2]. Acute changes are graded using a scale developed by the National Cancer Institute, ranging from faint erythema or dry desquamation to severe complications leading to death [1]. On the other hand, chronic changes may include dermal fibrosis, hyper- or hypopigmentation, telangiectasias, and dermal atrophy [1].

Despite the widespread use of radiation therapy in dermatology, there is currently no established standard of care for the treatment of radiation-induced skin changes [3]. Skin morphologies, such as dermal atrophy or dermatitis, occurring in sensitive areas like the face, can significantly impact patients' overall quality of life. Thus, it is imperative to explore and develop innovative procedures to enhance the treatment options for superficial radiation therapy (SRT)-induced skin changes. While most treatments currently employed are conservative in nature, such as skin hygiene and moisturizing, there have been case reports indicating beneficial results with the use of fractionated lasers [4]. In this paper, we present a case of a 70-year-old white male with squamous cell carcinoma in situ (SCCIS) on the right zygoma who underwent SRT, which subsequently developed a hypo-pigmented atrophic scar. The scar was treated successfully with a single session of Erbium:YAG laser therapy.

## Case presentation

**Figure 1:**
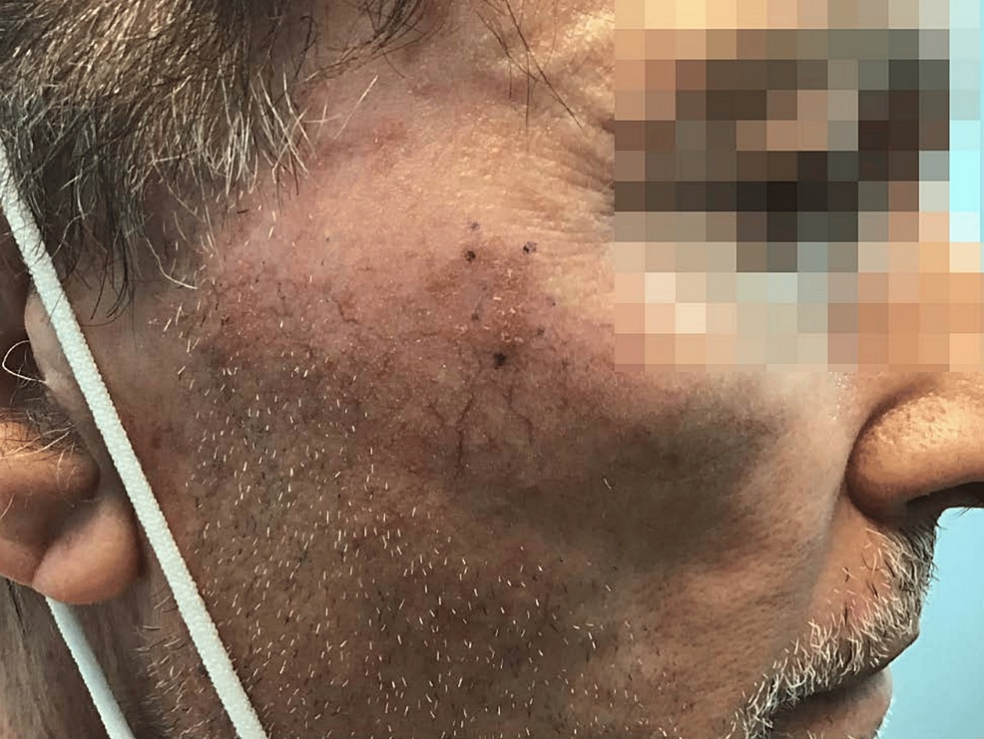
Area of squamous cell carcinoma in situ on the right zygoma

**Figure 2:**
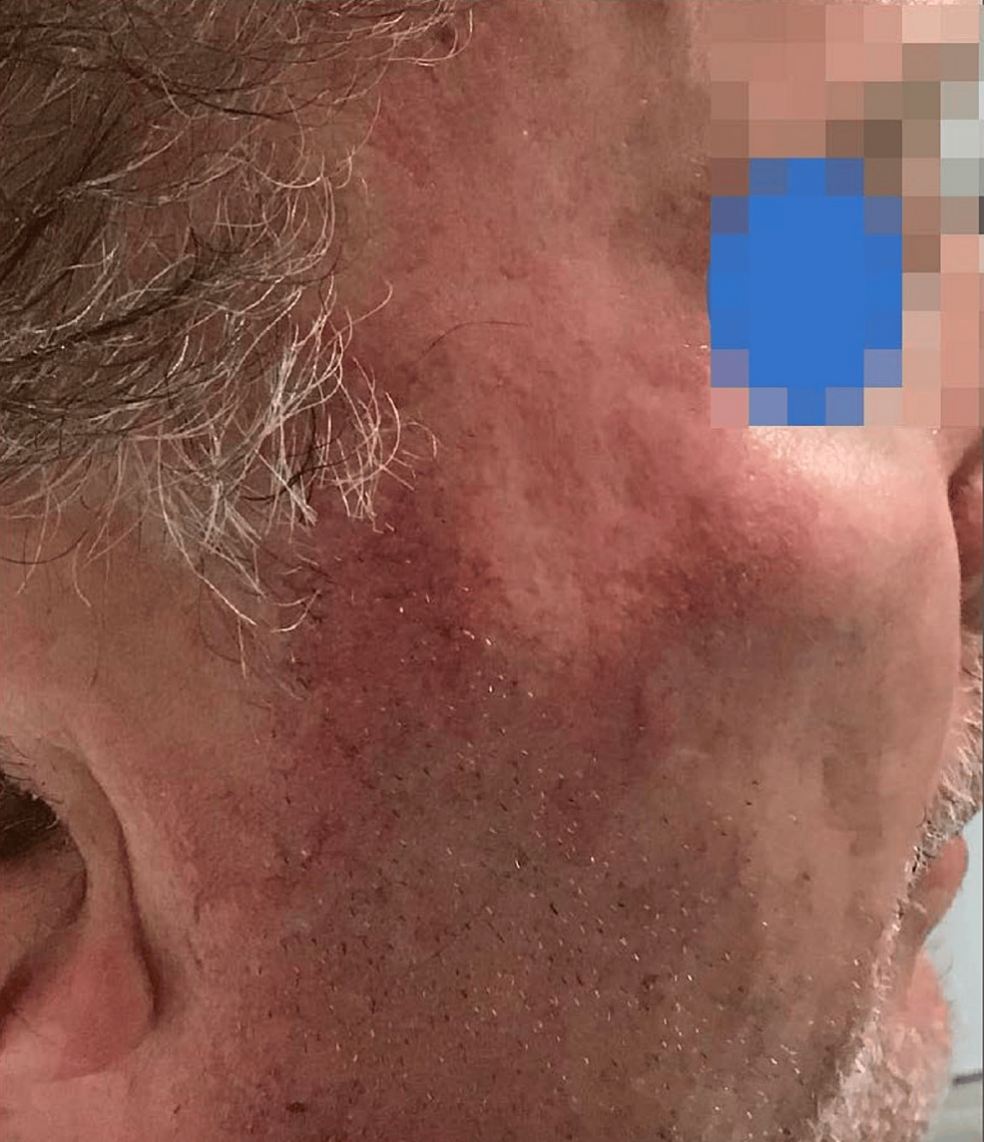
Atrophy and hypopigmentation of the right zygoma after SRT SRT, superficial radiation therapy

**Figure 3:**
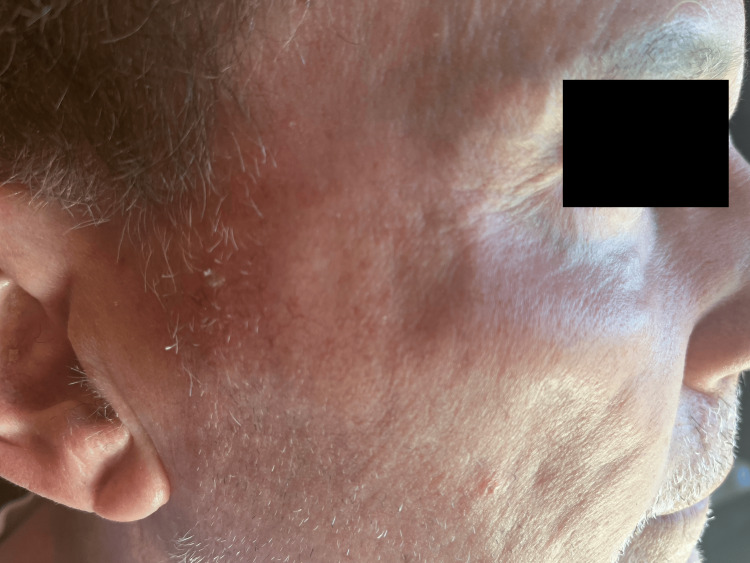
Improvement of the right zygoma after Erbium:YAG laser treatment

A 70-year-old white male presented to the dermatology clinic in August of 2021 with SCCIS on the right zygoma (Figure 1). After a discussion of the risks and benefits associated with the treatments, which included observation, topical 5-fluorouracil, cryosurgery, Mohs micrographic surgery, and SRT, the patient selected SRT. The lesion was treated with an energy setting of 70 kV, and the dose per fraction was 296.70 cGy. The cumulative dose of SRT was 5934.00 cGy, which was delivered over the course of 20 treatments. Aftercare instructions were given, such as treating the area with mild soap and avoiding sun exposure. In March 2023, the patient returned to the clinic seeking treatment for a hypopigmented atrophic scar on the right zygoma that developed due to the SRT (Figure 2). We discussed with the patient options for cosmetic improvement, which included broadband light treatment, fractionated Erbium:YAG laser treatment, and injection of poly-L-lactic acid (Sculptra). The patient elected to attempt Erbium:YAG laser treatment. The patient was informed that three laser therapy treatments would likely be required over the next three months, with the most significant improvement expected about three months after the final treatment. The Erbium:YAG laser treatment settings were 500 microns deep at 11% density, with fading around the perimeter of 250 microns to minimize the possibility of any lines of demarcation. The patient tolerated the laser treatment well and reported significant improvement after only four weeks, declining any further treatment (Figure 3).

## Discussion

The skin effects of radiation therapy are well documented, and their severity is graded on a scale of 1 to 5 by the National Cancer Institute (Table 1) [1]. These acute skin changes typically occur within 90 days of radiation therapy, with a second phase of progression possible 10-14 days later [1]. During the acute phase, radiation therapy induces transendothelial migration of leukocytes and immune cells to the radiation site, initiating an inflammatory reaction where various cytokines and chemokines act on endothelial cells, upregulating adhesion molecules [5]. Histopathologically, the acute changes are characterized by apoptotic keratinocytes, basal layer vacuolization, and edema [6].

**Table 1:** The graded scale from the National Cancer Institute for Radiation Dermatitis

Adverse event	Grade 1	Grade 2	Grade 3	Grade 4	Grade 5
Radiation dermatitis	Faint erythema or dry desquamation	Moderate to brisk erythema; patchy moist desquamation, mostly confined to skin folds and creases; moderate edema	Moist desquamation in areas other than skin folds and creases; bleeding induced by minor trauma or abrasion	Life-threatening consequences; skin necrosis or ulceration of full thickness dermis; spontaneous bleeding from involved site; skin graft indicated	Death

Chronic changes, on the other hand, may arise from an imbalance of proinflammatory and profibrotic cytokines, including tumor necrosis factor-alpha, interleukin 1 and 6, tumor growth factor-beta (TGF-B), and platelet-derived growth factor (PDGF) [4]. TGF-B and PDGF are responsible for fibroblast activation, while lymphocytic infiltration may impair vascularization and lead to fibrosis, atrophy, and necrosis [4]. Histopathologically, the chronic changes are characterized by eosinophilia, dermal collagen sclerosis, fibroblasts, and vascular alterations [6].

Typically, most patients delight in the aesthetic results of SRT, especially in well-vascularized areas where healing often occurs with minimal cosmetic imperfections. However, the chronic changes of extensive atrophy and hypopigmentation witnessed in this case are an unusual occurrence post-treatment. The proposed solutions for this patient included the use of fillers, such as poly-L-lactic acid (Sculptra), to stimulate collagen production or, alternatively, the application of fractionated Erbium laser, known for its capacity to induce collagen synthesis through controlled thermal injury. Currently, there is no established standard of care for radiation-induced skin changes. Previous research has suggested that conservative measures, including daily washing with soap and water, and application of a mid-potency steroid like mometasone furoate, along with the use of silver nylon dressings to cover the wound, may be beneficial [3]. Pharmacologic treatment options have shown promise, with small successful trials utilizing pentoxifylline in combination with vitamin E to reduce chronic fibrotic changes [4]. Laser therapy has also demonstrated positive results in improving hyperpigmentation and telangiectasias in reported cases [2]. Additionally, an ongoing pilot study suggests that fractionated laser therapy may reduce fibrosis and facilitate normal scar remodeling [4].

Our case adds to the growing evidence of the efficacy of fractionated laser therapy in treating SRT-induced dermal atrophy. By utilizing the Erbium:YAG laser treatment with settings of 500-micron depth at 11% density and 250 microns around the perimeter, we observed a noticeable improvement in atrophy within a mere four weeks.

## Conclusions

Radiation dermatitis is a common occurrence in patients undergoing radiotherapy, and it can lead to acute and chronic changes in the skin. Despite the lack of a standardized treatment approach, conservative measures, such as skin hygiene and moisturizing and pharmacologic options, have shown some benefits. This case report emphasizes the successful use of Erbium:YAG laser therapy in treating a hypo-pigmented atrophic scar resulting from SRT. The findings support the growing evidence that fractionated laser therapy holds promise for addressing radiation-induced skin changes, particularly dermal atrophy. Further research and larger studies are needed to establish the optimal protocols and effectiveness of laser therapy in managing radiation-induced skin alterations. Improved treatment options can significantly enhance patients’ quality of life by addressing the aesthetic and functional implications of these skin changes.
